# Fixed Points in Discrete Models for Regulatory Genetic Networks

**DOI:** 10.1155/2007/97356

**Published:** 2007-04-19

**Authors:** Dorothy Bollman, Omar Colón-Reyes, Edusmildo Orozco

**Affiliations:** 1 Departament of Mathematical Sciences, University of Puerto Rico, Mayaguez, PR 00681, USA; 2 Department of Computer Science, University of Puerto Rico, Río Piedras, San Juan, PR 00931-3355, USA

## Abstract

It is desirable to have efficient mathematical methods to extract information about regulatory iterations between genes from repeated measurements of gene transcript concentrations. One piece of information is of interest when the dynamics reaches a steady state. In this paper we develop tools that enable the detection of steady states that are modeled by fixed points in discrete finite dynamical systems. We discuss two algebraic models, a univariate model and a multivariate model. We show that these two models are equivalent and that one can be converted to the other by means of a discrete Fourier transform. We give a new, more general definition of a linear finite dynamical system and we give a necessary and sufficient condition for such a system to be a fixed point system, that is, all cycles are of length one. We show how this result for generalized linear systems can be used to determine when certain nonlinear systems (monomial dynamical systems over finite fields) are fixed point systems. We also show how it is possible to determine in polynomial time when an ordinary linear system (defined over a finite field) is a fixed point system. We conclude with a necessary condition for a univariate finite dynamical system to be a fixed point system.

## 

[1,2,3,4,5,6,7,8,9,10,11,12,13,14,15,16,17,18,19,20,21,22,23,24,25,26,27,28,29,30,31]
